# Supplementary material for: Analysis of SYK Gene as a Prognostic Biomarker and Suggested Potential Bioactive Phytochemicals as an Alternative Therapeutic Option for Colorectal Cancer: An In-Silico Pharmaco-Informatics Investigation
Source: J Pers Med. 2021 Sep 6;11(9):888. doi: 10.3390/jpm11090888 (PMC8470848; doi:10.3390/jpm11090888)
Supplement: Supplementary file 1 [file jpm-11-00888-s001.zip › jpm-1323900-supplementary.pdf]

Supplementary Table S1: Transcription levels of SYK gene between different subtypes of colorectal cancer and normal individuals using the Oncomine database.

Dataset	Colorectal Cancer Subtype	*P*-value	Fold change	*t*-test	Gene Rank (%)
**Graudens Colon**	Colorectal Carcinoma (18)	2.06E-5	2.451	5.122	5%
**Notterman Colon**	Colon Adenocarcinoma (18)	0.166	1.353	0.983	33%
**Gaspar Colon**	Colorectal Adenoma (56)	0.022	1.418	2.089	16%
**Ki Colon**	Colon Adenocarcinoma (50)	0.003	1.279	2.859	24%
**Kaiser Colon**	Rectosigmoid Adenocarcinoma (10)	0.001	1.365	3.695	16%
	Colon Adenocarcinoma (41)	4.03E-4	1.305	4.162	19%
	Cecum Adenocarcinoma (17)	0.001	1.517	3.497	19%
	Colon Mucinous Adenocarcinoma (13)	0.003	1.245	3.383	25%
	Rectal Adenocarcinoma (8)	0.185	1.116	0.942	49%
	Rectal Mucinous Adenocarcinoma (4)	0.308	1.080	0.546	58%
**Hong Colorectal**	Colorectal Carcinoma (70)	1.21E-5	1.713	5.526	17%
**Sabates-Bellver Colon**	Rectal Adenoma (7)	8.02E-4	1.436	4.366	19%
	Colon Adenoma (25)	9.28E-6	1.468	4.718	21%
**Kurashina Colon**	Colon Mucinous Adenocarcinoma (4)	0.129	1.067	1.387	24%
	Rectal Adenocarcinoma (38)	0.009	1.036	2.463	31%

	Colon Adenocarcinoma (52)	0.334	1.005	0.432	56%
**Skrzypczak Colorectal 2**	Colon Carcinoma (5)	0.002	1.235	3.784	27%
	Colon Carcinoma (5)	0.342	1.044	0.420	50%
	Colon Adenoma (5)	0.721	-1.051	-0.603	77%
	Colon Adenoma (5)	0.779	-1.082	-0.811	78%
**Skrzypczak Colorectal**	Colorectal Adenocarcinoma (45)	0.005	1.127	2.625	31%
	Colorectal Carcinoma (36)	0.618	-1.017	-0.300	70%
**TCGA Colorectal**	Rectal Adenocarcinoma (60)	4.92E-4	1.233	3.430	31%
	Colon Adenocarcinoma (101)	0.033	1.102	1.864	44%
	Cecum Adenocarcinoma (22)	0.093	1.108	1.351	47%

Supplementary Table S2: Differential expression pattern analysis of SYK in Colorectal adenocarcinoma (COAD) based on different variables and different stages where the comparison was made between normal vs different stages of cancer using UALCAN database. *P*-value in bold denotes statistically significant values.

Variables	Different stages	Comparisons	N	Statistical significance
Sample types	Normal		41	
	Primary tumor	Normal-Vs-Primary	286	**6.154500E-04**
Individual cancer stages	Stage1	Normal-Vs-Stage1	45	**3.144300E-02**
	Stage2	Normal-Vs-Stage2	110	**1.092400E-02**
	Stage3	Normal-Vs-Stage3	80	**3.619600E-02**
	Stage4	Normal-Vs-Stage4	39	**2.325700E-02**
Patient’s race	Caucasian	Normal-Vs-Caucasian	193	**1.036450E-02**

	African-American<br>Asian	Normal-Vs-African American<br>Normal-Vs-Asian	55<br>11	**1.156890E-03**<br>4.710000E-01
Patient's gender	Male<br>Female	Normal-Vs-Male<br>Normal-Vs-Female	156<br>127	**7.161000E-04**<br>**1.651970E-02**
Patient's weight	Normal weight<br>Extreme weight<br>Obese<br>Extreme obese	Normal-Vs-Normal Weight<br>Normal-Vs-Extreme Weight<br>Normal-Vs-Obese<br>Normal-Vs-Extreme Obese	70<br>74<br>56<br>10	**9.699300E-03**<br>**2.808200E-02**<br>**4.997900E-03**<br>3.058600E-01
Patient's age	21-40 Yrs.<br>41-60 Yrs.<br>61-80 Yrs.<br>81-100 Yrs.	Normal-Vs-Age(21-40Yrs)<br>Normal-Vs-Age(41-60Yrs)<br>Normal-Vs-Age(61-80Yrs)<br>Normal-Vs-Age(81-100Yrs)	12<br>90<br>149<br>32	5.087000E-01<br>**1.117900E-02**<br>**4.171100E-04**<br>2.810000E-01
Histological subtype	Adenocarcinoma<br>Mucinous adenocarcinoma	Normal-Vs-Adenocarcinoma<br>Normal-Vs-Mucinous-adenocarcinoma	243<br>37	**1.767880E-04**<br>1.519230E-01
Nodal metastasis status	NO<br>N1<br>N2	Normal-Vs-N0<br>Normal-Vs-N1<br>Normal-Vs-N2	166<br>70<br>47	**1.558190E-03**<br>**3.055900E-03**<br>1.855820E-01
TP53 mutation status	TP53-Mutant<br>TP53-NonMutant	Normal-Vs-TP53-Mutant<br>Normal-Vs-TP53-NonMutant	160<br>122	**1.972550E-04**<br>8.074100E-02

Supplementary Table S3: Promoter methylation level of SYK in Colorectal adenocarcinoma (COAD) based on different variables and in a comparison between normal vs different stages of cancer from UALCAN database. *P*-value in bold denotes statistically significant values**.**

Variables	Different stages	Comparisons	N	Statistical significance
Sample types	Normal		37	
	Primary tumor	Normal-Vs-Primary	313	**8.576500E-03**
Individual cancer stages	Stage1	Normal-Vs-Stage1	50	**1.113830E-02**
	Stage2	Normal-Vs-Stage2	122	**1.955640E-02**
	Stage3	Normal-Vs-Stage3	88	**2.920500E-02**
	Stage4	Normal-Vs-Stage4	41	2.522800E-01
Patient's race	Caucasian	Normal-Vs-Caucasian	220	**5.938700E-03**
	African-American	Normal-Vs-African American	61	7.700000E-02
	Asian	Normal-Vs-Asian	11	**8.001300E-03**
Patient's gender	Male	Normal-Vs-Male	167	**1.099520E-02**
	Female	Normal-Vs-Female	144	**1.667910E-02**
Patient's age	21-40 Yrs	Normal-Vs-Age(21-40Yrs)	13	8.981600E-01
	41-60 Yrs	Normal-Vs-Age(41-60Yrs)	96	**2.301900E-02**
	61-80 Yrs	Normal-Vs-Age(61-80Yrs)	165	**2.190300E-02**
	81-100 Yrs	Normal-Vs-Age(81-100Yrs)	37	**1.639220E-03**
Patient's weight	Normal Weight	Normal-Vs-Normal Weight	83	**1.195270E-03**
	Extreme Weight	Normal-Vs-Extreme Weight	80	1.161640E-01
	Obese	Normal-Vs-Obese	66	**1.758360E-02**
	Extreme obese	Normal-Vs-Extreme Obese	12	1.501010E-01
Nodal metastasis status	NO	Normal-Vs-N0	185	**8.704600E-03**
	N1	Normal-Vs-N1	74	1.622990E-01
	N2	Normal-Vs-N2	50	**1.919770E-02**
Tumor histology	Adenocarcinoma	Normal-Vs-Adenocarcinoma	264	**1.065780E-02**
	Mucinous adenocarcinoma	Normal-Vs-Mucinous adenocarcinoma	44	**4.724300E-02**

TP53 Mutation status	TP53 Mutant	Normal-Vs-TP53 Mutant	174	**1.668120E-02**
	TP53 NonMutant	Normal-Vs-TP53 NonMutant	136	**9.900800E-03**

Supplementary Table S4: Positively correlated genes of SYK in COAD

GEPIA		UALCAN	
**Gene Symbol**	**PCC**	**Gene Symbol**	**PCC**
SECISBP2	0.59	SECISBP2	0.61
PCDH19	0.55	RNF20	0.54
CCDC170	0.52	TSTD2	0.53
RNF20	0.51	SNX30	0.51
EFCAB14	0.50	ZNF782	0.51
NAA35	0.50	C6orf97	0.51
CDC14B	0.49	KIAA0494	0.5
AXIN2	0.49	CDC14B	0.5
TSTD2	0.49	GLE1	0.49
SMC5	0.48	NAA35	0.49
TMEM8B	0.48	SMC5	0.49
		TCF7	0.49
		TBC1D13	0.48
		FKTN	0.48
		FGGY	0.48
		GOLGA1	0.48

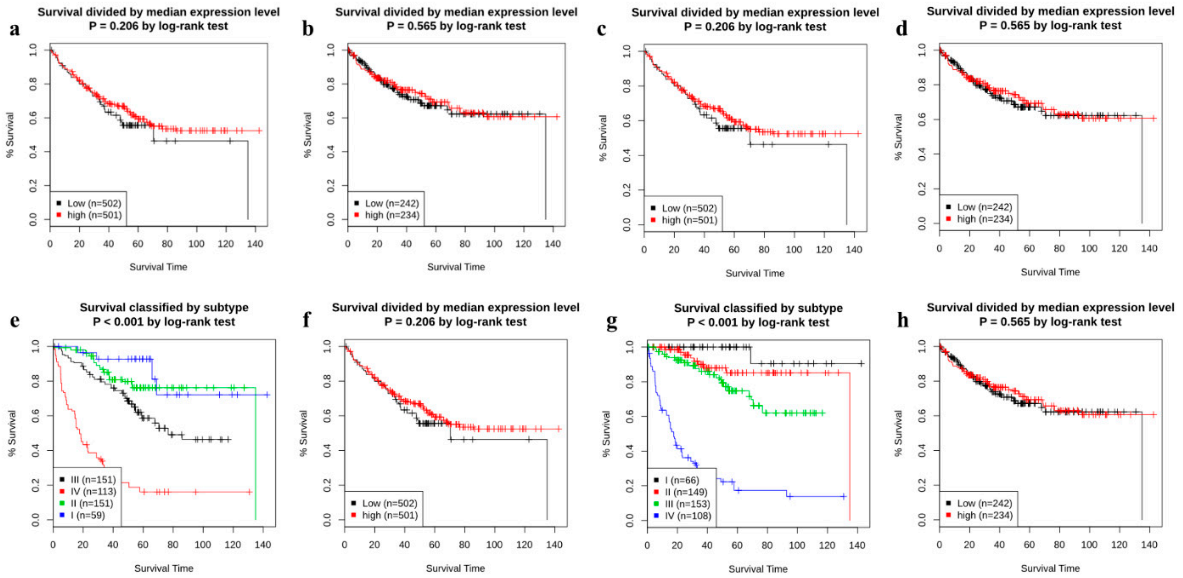

Supplementary Figure S1: Prognostic value of SYK gene in human colorectal cancer from GENT2 database. (a) KM-plots divided by median cut-off- overall survival; (b) KM-plots divided by median cut-off- diseases specific survival; (c) KM-plots divided by median cut-off- overall survival for DUKE_Stage; (d) KM-plots divided by median cut-off- diseases specific survival for DUKE_Stage; (e) KM-plots divided by subtypes- overall survival for AJCC_Stage (f) KM-plots divided by median cut-off- overall survival for AJCC_Stage; (g) KM-plots divided by subtypes- diseases specific survival for AJCC_Stage; (h)) KM-plots divided by median cut-off- diseases specific survival for AJCC_Stage.

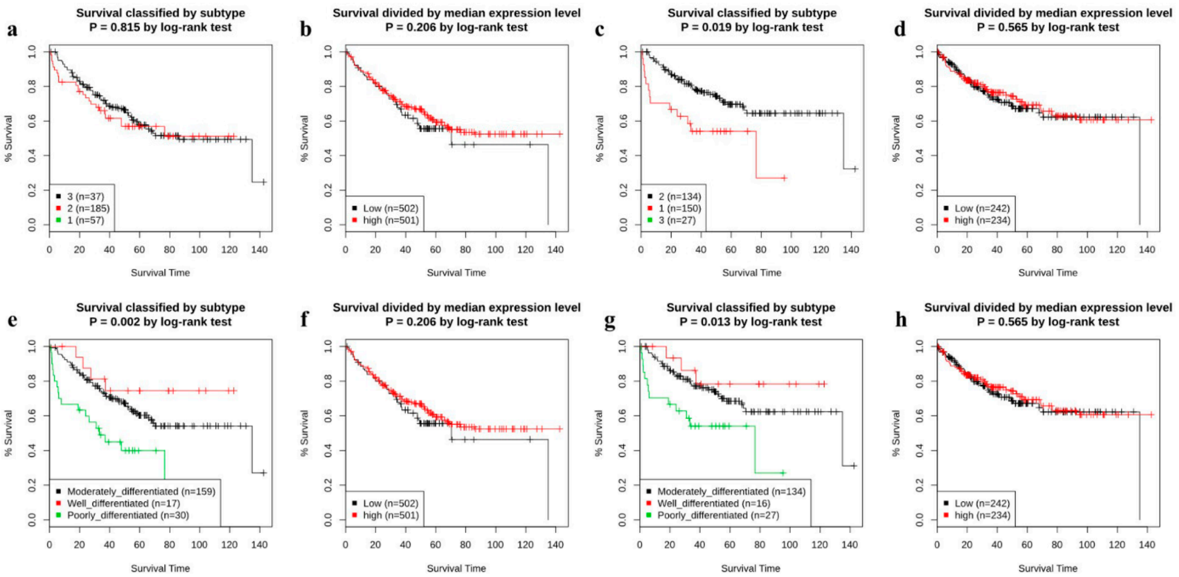

Supplementary Figure S2: Prognostic value of SYK gene in human colorectal cancer from GENT2 database. (a) KM-plots divided by subtypes- overall survival for Grade; (b) KM-plots divided by median cut-off- overall survival for Grade; (c) KM-plots divided by subtypes- diseases specific survival for Grade; (d) KM-plots divided by median cut-off- diseases specific survival for Grade; (e) KM-plots divided by subtypes- overall survival for Histology; (f) KM-plots divided by median cut-off- overall survival for Histology; (g) KM-plots divided by subtypes- diseases specific survival for Histology; (h) KM-plots divided by median cut-off- diseases specific survival for Histology.

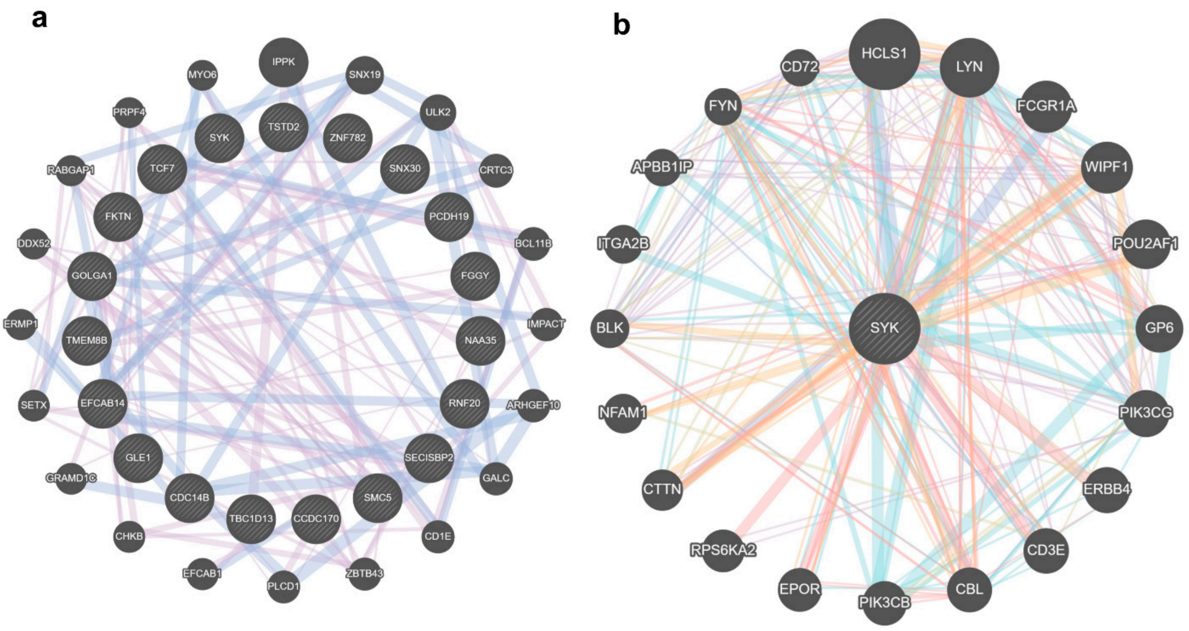

Supplementary Figure S3: Protein-protein interaction network. (a) Interaction network of SYK with the positively correlated genes of COAD; (b) interaction network of SYK with the significantly interacted gene based on co-expression, genetic interactions, pathways, physical interactions, co-localization and predicted shared protein domains.
